# The Impact of Mobile Health (mHealth) Apps on Gestational Diabetes: A Systematic Review

**DOI:** 10.7759/cureus.79375

**Published:** 2025-02-20

**Authors:** Paraskevi Giaxi, Victoria Vivilaki, Maria Iliadou, Ermioni Palaska, Athina Diamanti, Kleanthi Gourounti

**Affiliations:** 1 Department of Midwifery, University of West Attica, Athens, GRC

**Keywords:** diabetes gestational, intelligent systems, mobile apps (mhealth), pregnancy, women

## Abstract

Gestational diabetes mellitus (GDM) poses a significant health risk for pregnant women, as it is associated with an increased likelihood of developing type II diabetes and cardiovascular complications. In addition, there is a high risk for pregnancy-related complications, emphasizing the need for effective management of GDM. This study aimed to investigate the potential impact of mHealth applications on gestational diabetes-related outcomes.

A systematic review was conducted, following the Preferred Reporting Items for Systematic Reviews and Meta-Analyses (PRISMA) guidelines. The search was conducted in Pubmed, Scopus, and Web of Science, using the following search terms: (smartphone* OR mobile OR mHealth OR “mobile health”) AND (“gestational diabetes” OR “maternal diabetes” OR “pregnancy diabetes” OR “pregnancy-induced diabetes” OR “perinatal diabetes”). In order to be included in the systematic review, the studies had to be papers published in peer-reviewed journals, published from February 2, 2020 to February 2, 2025, in the English language, being randomized trials, evaluating the impact of mHealth apps on women with GDM, excluding studies that incorporated additional technological interventions (e.g., WeChat supportive groups). The extracted data were the following: authors, country, total and per group number of participants, participants’ characteristics, interventional content, assessments, time of the assessments, results, and potential information about participant adherence. The Jadad Scale was used for quality evaluation.

Six studies met the predefined criteria and were included in the systematic review. Three studies found no or minimal benefits from the use of such applications. One study demonstrated highly significant benefits, not only in the management of GDM but also in reducing pregnancy-related complications. Another study indicated greater adherence to treatment and fewer admissions to neonatal intensive care units, while a third study reported a shift toward better postpartum health behaviors, which was closely associated with the use of mHealth applications, suggesting that those apps could support women in adopting healthier habits after childbirth. All studies reporting positive outcomes were carried out in Eastern Asian Countries (Singapore and China), whereas studies conducted in Europe found minimal or no benefits. The score on the Jadad Scale ranged from 2 to 3, indicating moderate to low quality. Οverall, while some promising findings were observed, further research is essential to evaluate these apps more thoroughly and enhance their effectiveness.

Greater culturally influenced adherence to healthcare instructions leads to more significant benefits for Asian women with GDM. Cultural factors should be carefully considered when designing and implementing mHealth applications for women with GDM.

## Introduction and background

Gestational diabetes mellitus (GDM) is associated with multiple adverse health outcomes for women and neonates. A meta-analysis of eight studies, encompassing a total of 276,829 participants, found that women with a history of GDM were 13.2 times more likely to develop type 2 diabetes compared to those who had no prior history [1]. Similarly, a meta-analysis of nine studies with a total sample of 5,390,591 women, reported that those with GDM had a 1.98-fold increased risk of developing cardiovascular disease compared to those without GDM [2]. Additionally, GDM has been associated with an increased risk of several types of cancer, including breast, liver, stomach, and thyroid cancer [3]. These findings highlight the substantial long-term health risks associated with GDM, underlining the urgent need for effective interventions to manage and mitigate its impact.

If not properly managed, GDM can lead to significant complications for both the mother and the newborn. One of the primary maternal complications is the increased risk of hypertension and pre-eclampsia. Women with gestational diabetes are more likely to develop these conditions during pregnancy [4,5]. Pre-eclampsia is a severe disorder characterized by high blood pressure and organ dysfunction, which can result in serious complications for both the mother and the newborn if not properly managed. This underscores the importance of developing health promotion programs to minimize the consequences of GDM [4].

Regarding neonatal outcomes, one of the most significant complications is macrosomia. Infants born to mothers with uncontrolled GDM are at an increased risk of being large for gestational age which increases the risk of complications during delivery, including a greater need for cesarean section [6,7]. Additionally, macrosomic newborns are more susceptible to birth-related injuries [8]. Another major concern is neonatal hypoglycemia, a condition in which newborns experience low blood sugar levels shortly after birth [9]. This occurs because elevated maternal blood glucose levels during pregnancy stimulate excessive fetal insulin production. To prevent complications, careful monitoring and appropriate medical management are essential for stabilizing blood sugar levels in affected newborns [10]. Additionally, GDM-related complications are respiratory distress syndrome, which is more prevalent in preterm infants born to mothers with GDM. This condition impairs the newborn's ability to breathe independently, posing a serious health risk [11]. Another frequent complication is neonatal jaundice, characterized by a yellowish discoloration of skin and screla, typically resulting from excessive urobilinogen production or impaired bilirubin excretion. Infants born to mothers with GDM are at higher risk of developing this condition [12].

GDM poses a risk to both pregnant mothers and their newborns, hence it is necessary to design and execute effective health promotion initiatives to limit its impact [13]. Hence, this study focused on the role of mobile health (mHealth), which involves the use of mobile technologies, such as smartphones and wireless devices, to enhance healthcare services, facilitate remote monitoring, improve disease management, and support patient education [14,15]. mHealth applications offer significant benefits to patients by helping them make informed health decisions, adhere to treatment plans and improve their overall well-being [16]. Additionally, mHealth applications serve as a valuable source of information, educating patients about their health and improving communication with healthcare professionals [17]. In that context, the aim of the present study was to investigate the potential impact of mHealth applications on the management of GDM. Specifically, the study explored the effects of these interventions on both women diagnosed with GDM as their neonates.

## Review

Materials and methods

Literature Search

A systematic literature search was conducted for English-language randomized controlled trials (RCTs) published between February 2, 2020 and February 2, 2025. The search was performed in Pubmed, Scopus, and Web of Science, using the keywords: (smartphone* OR mobile OR mHealth OR “mobile health”) AND (“gestational diabetes” OR “maternal diabetes” OR “pregnancy diabetes” OR “pregnancy-induced diabetes” OR “perinatal diabetes”). To ensure comprehensive coverage, a snowball sampling approach was applied to identify relevant studies that may have been missed. This involved reviewing issues of related journals, examining reference lists of included studies, and searching other relevant publications. The selection process adhered to the Preferred Reporting Items for Systematic Reviews and Meta-Analyses (PRISMA) guidelines for systematic reviews [18]. The literature search process was carried out by the first, second, and third authors.

Study Selection

For study selection, the inclusion criteria required: (1) original research articles published in peer-reviewed journals, (2) RCTs with a clearly defined intervention and control group, (3) intervention based exclusively on mHealth application, and (4) participants diagnosed with GDM. Studies were excluded (1) if they combined the mHealth application with other technological interventions (e.g., web-based interventions accessed via personal computers) and (2) if not published in English. The identified abstracts were organized using Zotero reference management software. The study selection process was carried out by the first and the second authors, with any discrepancies resolved by the fourth and fifth authors.

Data Extraction

The extracted data were the following: author(s), country, total and per group number of participants, participants’ characteristics, interventional content, assessment measures, timing of assessments, results, and potential information about participant adherence. The first, second, and third authors performed the data extraction independently, with any disagreements resolved by other authors.

Quality Evaluation

The quality of the trials was assessed using the Jadad Scale. The Jadad Scale is a short evaluation tool with a score range of 0 to 5 points, designed to assess trial quality. It allocates two points for randomization, two points for double blinding, and one point for reporting reasons for withdrawals and dropouts [19]. The first, second, and third authors performed the data extraction independently, with any disagreements resolved by other authors.

Results

Flow of Information

**Figure 1:**
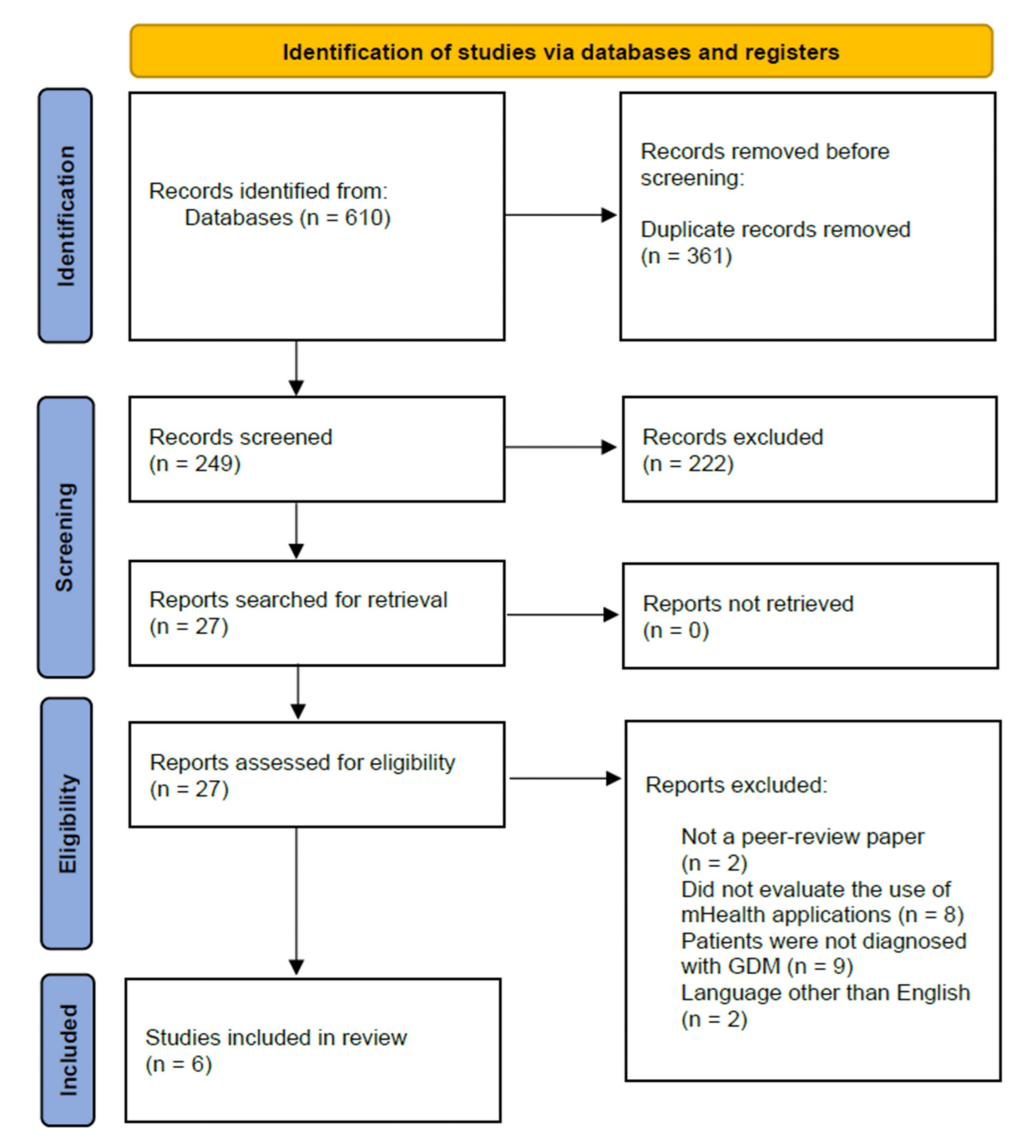
Selection process for the included studies

The search process yielded a total of 610 potentially relevant records. After duplicate removal, 249 original records remained. Of those, 222 were excluded during the initial screening phase for obvious reasons (e.g., explicitly stating in the title that they were protocol studies for future RCTs). The remaining 27 full-text articles were assessed for eligibility based on the predefined inclusion and exclusion criteria. Of those, 21 did not meet at least one of the pre-set criteria and were subsequently excluded. As a result, six studies met all eligibility criteria and were included in the systematic review (Figure 1).

Summary of the Included Studies

In order to minimize the risk of bias while reporting the study results, they are reported by the order they were found and included in the systematic review (Table 1). The first study found in the literature was carried out by Yew et al. [20] in Singapore, aiming to evaluate the effects of a smartphone application-based lifestyle coaching program (Habits-GDM) on gestational weight gain, glycemic control, and maternal and neonatal outcomes among women diagnosed with GDM. A total of 340 pregnant women diagnosed with GDM between 12 and 30 weeks of gestation were equally randomized to the intervention and the usual care group. The intervention included automated lifestyle coaching, dietary guidance, glucose and weight monitoring, and behavioral modification support. The study assessments carried out at baseline and 35-37 weeks of gestation, included glycemic control (blood glucose levels and self-monitoring frequency), maternal weight changes, delivery outcomes, and neonatal complications. Lower mean glucose levels (mean difference = -0.15 mmol/L, p=0.011) and fewer instances of glucose exceeding target levels (premeal: 17.9% vs. 23.3%, p=0.003; two-hour post-meal: 19.9% vs. 50%, p<0.001) were noted in the intervention group compared to controls. Additionally, the intervention group was associated with a significant reduction in composite neonatal complications (38.1% vs. 53.7%, p=0.006). No significant differences were found between groups for maternal hypertensive disorders, mode of delivery, insulin use, or weight gain.

**Table 1:** Main findings of the reviewed studies SPAROW: Smartphone App to Restore Optimal Weight

Authors	Country	Assessments	Intervention details	Main results
Yew et al. (2021) [20]	Singapore	Baseline and 35-37 weeks: Glycemic control, maternal weight changes, delivery outcomes, neonatal complications	Habits-GDM: Automated lifestyle coaching, dietary guidance, glucose and weight monitoring, behavioral modification support	Lower mean glucose levels, reduced glucose target exceedance, lower neonatal complications; no significant differences in maternal weight gain, delivery mode, insulin use
Khunti et al. (2023) [21]	United Kingdom	Baseline, 6 months, 12 months: Physical activity, self-reported exercise self-efficacy, anxiety, depression, quality of life	Hybrid intervention: Two group-based education sessions, mobile app for activity tracking, goal setting, automated feedback, peer support	No significant change in physical activity; improved exercise self-efficacy, lower anxiety, improved quality of life
Potzel et al. (2022) [22]	Germany	Baseline and 6 months: Adherence to lifestyle goals, insulin sensitivity, glucose tolerance, body composition, psychological well-being	TRIANGLE: Habit-based behavior change strategies, interactive challenges, chat-based coaching, knowledge library	No significant differences in lifestyle goal adherence; control group had higher fiber intake and better health habits; app engagement decreased over time
Lim et al. (2021) [23]	Singapore	Baseline, 6 weeks, 4 months postpartum: Weight restoration, dietary intake, metabolic markers, physical activity, quality of life	SPAROW: Logging weight, meals, and activity levels, real-time feedback from dietitians, physiotherapists, occupational therapists	No significant weight restoration effect; lower caloric intake, improved health behaviors, increased step counts, higher emotional distress
Garnweidner-Holme et al. (2020) [24]	Norway	Baseline and 36 weeks: Dietary habits through Healthy Dietary Score for Pregnant+ (HDS-P+)	Pregnant+ App: Dietary guidance and glucose monitoring for GDM-specific dietary recommendations	No significant effects on dietary behavior
Zhuo et al. (2022) [25]	China	Baseline and 12 weeks postpartum: Medication adherence, insulin injection technique, glycemic control, pregnancy and neonatal outcomes	Clinical pharmacist-led app: Medication adherence, insulin injection guidance, glycemic control monitoring	Higher medication adherence, improved injection proficiency, better glycemic control, fewer hypoglycemic events, lower neonatal ICU admissions; higher cesarean delivery rate

Another RCT was conducted by Khunti et al. [21] in the United Kingdom, to evaluate the effectiveness of a hybrid lifestyle intervention combining structured group education with a mHealth component in promoting physical activity among women with a history of GDM. A total of 293 women (mean age: 35.1 years; 40% from ethnic minority backgrounds) with prior GDM were recruited from two hospitals and randomly assigned to either routine care (n=150) or a hybrid intervention group (n=143). The intervention consisted of two group-based education sessions, where participants received lifestyle modification guidance, were encouraged to engage in an additional 30 minutes of moderate activity per day, and were provided with an activity tracker. A mobile application was used, enabling the study participants to track their activity levels, set goals, receive automated feedback, and engage in peer support. The measurements regarding physical activity were carried out at baseline, six months, and 12 months. More specifically, the participant's physical activity levels were objectively measured using the GENEActiv accelerometer (ActivInsights, Cambs, UK). This was worn on the non-dominant wrist for eight consecutive days, recording average daily acceleration (mg), with valid data requiring at least 16 hours/day of wear time, and processed using the GGIR R-package (Medical Research Council, Swindon, UK) for calibration and non-wear detection. Secondary outcomes included self-reported exercise self-efficacy, anxiety and depression, and health-related quality of life. There was no significant improvement in overall physical activity levels at 12 months, with a between-group difference of 0.95 mg (95% CI: -0.46 to 2.37, p=0.185), which equates to an increase of approximately 500 steps per day. Yet, the intervention group participants had higher self-efficacy for exercise (0.54, 95% CI: 0.05 to 1.02, p=0.029), lower anxiety levels (-0.91, 95% CI: -1.74 to -0.09, p=0.031), and improved quality of life (0.05, 95% CI: 0.004 to 0.09, p=0.032) compared the control group participants of the study.

Another related RCT was carried out by Potzel et al. [22] in Germany to evaluate the effectiveness of TRIANGLE, a smartphone-based lifestyle intervention designed to modify cardiometabolic risk behaviors in women with GDM, three to 18 months postpartum. The intervention aimed for the participants was 150 minutes/week of moderate-to-high intensity physical activity, dietary fiber intake ≥15 g/1,000 kcal, fat intake <30% of total energy, saturated fat intake <10%, and weight loss of ≥5% (if BMI ≥23 kg/m²) or weight maintenance (if BMI <23 kg/m²). The measurements were carried out at baseline and after a six-month period, evaluating adherence to those targets. Insulin sensitivity, glucose tolerance, body composition, and psychological well-being were also assessed. Thirty-three participants were randomized to the intervention group and an equivalent number to the usual care group. The intervention lasted six months and used habit-based behavior change strategies, interactive challenges, chat-based coaching, and a knowledge library. No significant differences were noted between the two groups regarding the number of goals achieved. In general, the only significant differences in favor of the control group were higher fiber intake (p=0.007) and better health-related habits (p=0.001). It is also important to note that engagement with the app had a decreasing trend over time, with higher dropout rates among non-native German speakers.

An additional related study was conducted by Lim et al. [23] in Singapore to evaluate the effectiveness of the Smartphone App to Restore Optimal Weight (SPAROW) in promoting postpartum weight loss among women with a recent history of GDM. More specifically, the aim was to help women return to their first-trimester weight or achieve a ≥5% weight reduction (if first-trimester BMI >23 kg/m²) at four months postpartum. One hundred and one women were randomly assigned to the intervention and 99 to the standard care control group. The app allowed participants to log their weight, meals, and activity levels and provided real-time feedback from dietitians, physiotherapists, and occupational therapists. The assessments of this trial were performed at baseline, six weeks, and four months postpartum. The primary assessments focused on weight restoration and reduction and the secondary on dietary intake, metabolic markers, physical activity, and quality of life. No significant differences were noted in achieving optimal weight. However, the intervention group reported significantly lower caloric intake (p<0.001), improved health-directed behaviors (p=0.045), and increased step counts (p=0.04), while experiencing higher emotional distress scores (p=0.01), possibly as a result of higher awareness for the risks of GDM. The engagement of users was sustained at 60.8% at four months, a considerably high proportion.

A further study was conducted by Garnweidner-Holme et al. [24]. This RCT was carried out in Norway to assess the effectiveness of the Pregnant+ smartphone application. The application aimed to provide dietary guidance and glucose monitoring, to support adherence to GDM-specific dietary recommendations. Two hundred and thirty-eight women with GDM were recruited from five related clinics in Oslo and randomized to an intervention (n=115) and a usual care control group (n=123). Glucose monitoring features and related dietary recommendations were included in the app. Participants’ dietary habits were assessed at baseline and 36 weeks of gestation, through the Healthy Dietary Score for Pregnant+ (HDS-P+). The analysis between the two groups leads to non-significant effects.

The final study included in the present systematic review was that of Zhuo et al. [25]. This RCT was carried out in China, testing the efficacy of a clinical pharmacist-led smartphone application to improve medication adherence, insulin injection technique, and diabetes-related outcomes in a sample of women receiving insulin therapy. Half of the sample was randomly assigned to the intervention and half to the control group, with follow-up extending until 12 weeks postpartum. The mobile app targeted medication adherence and injection technique use, which were the primary outcomes, while the secondary outcomes were injection proficiency, insulin requirements, glycemic control before and after delivery, hypoglycemia incidence, and pregnancy and neonatal outcomes. The intervention group had significantly higher medication adherence (69.0% vs. 34.4%, p<0.001). In addition, the intervention group participants had higher injection proficiency, lower preprandial insulin dosage, improved prepartal fasting plasma glucose and two-hour postprandial glucose levels, better puerperal glycemic control, less frequent hypoglycemic events, and reduced neonatal intensive care unit admissions (all p<0.05).

Quality Evaluation

The scoring of the studies on the Jadad Scale is presented in Table 2. The score on the Jadad Scale ranged from 2 to 3. In general, the inability to create double-blinding conditions due to the nature of the intervention debarred reaching higher scores.

**Table 2:** Jadad Scale scoring

Study	Randomized	Appropriate randomization	Double-blind	Blinding method appropriate	Withdrawals/dropouts reported	Total score
Garnweidner-Holme et al. (2020) [24]	1	1	0	0	1	3
Khunti et al. (2023) [21]	1	1	0	0	1	3
Lim et al. (2021) [23]	1	1	0	0	1	3
Potzel et al. (2022) [22]	1	1	0	0	0	2
Yew et al. (2020) [21]	1	1	0	0	0	2
Zhuo et al. (2022) [25]	1	0	0	0	1	2

Discussion

The above research leads to several findings regarding the impact of mobile phone applications on women with GDM. Khunti et al. [21], Potzel et al. [22], and Garnweidner-Holme et al. [24] found either minimal or no benefits. Yew et al. [20] demonstrated highly significant benefits, including improved glycemic control and reduced neonatal complications. Two studies yielded inconclusive but promising findings. Specifically, Zhuo et al. [25] found greater medication adherence and fewer NICU admissions, whereas Lim et al. [23] reported better postpartum health behaviors, but also an increase in psychological distress among women taking part in the intervention.

Regarding the quality of evidence, it was rated as medium to low according to the Jadad Scale, with scores ranging from 2 to 3 points out of 5. Notably, none of the studies received a quality rating of 5 or even 4. This limitation is mainly attributed to the lack of double-blinding, which was noted in all studies. In addition, reporting of withdrawals and dropouts varied between the studies included. For all the above reasons, the overall reliability of the findings is considered to be low to moderate.

Looking critically at the studies’ findings, a key point of interest also relates to the potential negative effects found in some of the studies in this systematic review. The study by Lim et al. [23] recorded an increase in levels of psychological distress (distress), while the study by Zhuo et al. [25] indicated an increase in cesarean sections in the intervention group. Presumably, these findings are attributable to an increased awareness of the risks associated with gestational diabetes, which may lead women to higher levels of stress and subsequent adaptive decisions to reduce the risk they face. Consequently, it seems imperative that relevant mobile phone applications incorporate stress management techniques, such as relaxation exercises, which are particularly helpful in improving mental health in women with gestational diabetes [26].

A second suggestion for those who develop related applications concerns the linguistic adaptation of these applications. The study by Potzel et al. [22] in Germany found that dropout of the intervention was more frequent among women who did not have German as their mother tongue. It is therefore required that these apps are translated into multiple languages, but also culturally adapted. Indeed, nutrition during gestational diabetes is affected by cultural parameters, as women from different backgrounds have different dietary preferences [27]. Therefore, in addition to language adaptation, cultural adaptation of the content of these apps is required to make them more effective and appropriate for the needs of different populations.

An additional suggestion, although not directly derived from the findings of this systematic review, concerns the personalization of applications through the use of artificial intelligence (AI), which is one of the main technological advances in Industry 4.0 [28]. It is undeniable that suggestions are needed to further improve the effects observed through these applications, as even in studies where significant benefits were found, such as the study by Yew et al. [20], some parameters were not improved. The use of AI can help personalize the content of applications, allowing applications to learn from the profiles and general behaviors of users when using them [29]. The integration of AI technologies is therefore required to optimize the effects of these applications, which is a key proposal for future research.

In addition, future research should focus more on the effects on the children of women with gestational diabetes. Existing studies have been mainly short-term oriented and have not examined long-term effects on children. For example, gestational diabetes has been associated with adverse outcomes in later development, such as an increased likelihood of autism [30]. What is needed, therefore, is more research examining the long-term health effects of using these apps on children of women with gestational diabetes.

Finally, this systematic review faces the limitations of the few studies analyzed (N=6), the use of specific databases, and the study of research published exclusively in English. Therefore, it is imperative to conduct additional systematic reviews that include studies from databases in different languages, such as Chinese, in order to examine more studies and to gain a more complete picture of the effects of using such apps by women with gestational diabetes. A comprehensive understanding of the effects of these apps will further enable the formulation of more robust recommendations for clinical practice.

## Conclusions

The question about the effects of applications focusing on the treatment of gestational diabetes could not be answered simplistically, i.e., a “yes and no” answer could not be given. The development of technological applications does not negate the fact that the ultimate recipient of these applications is the patient, who, as a human, is affected by several different factors influencing the acceptance, use, and ultimately the outcomes of the applications. Indeed, the use of such applications could in general be justified and considered useful, as could any effort to promote the health of women with gestational diabetes, which is expected to have positive effects on them and their children.

This systematic review highlights a particularly important factor that differentiates the impact of these applications, of the cultural context. For this reason, studies in Asia, where there is likely to be higher compliance with health professional guidelines and higher health literacy, particularly in countries such as Singapore, lead to more significant benefits than studies in European countries. In addition, differentiation by cultural context appears to exist within countries themselves, where it is found that the use of these applications is more difficult for women from non-dominant cultural groups. Particular emphasis and weight should therefore be given to the linguistic and cultural adaptation of these applications to their target audience, maximizing the benefits involved. At the same time, it is necessary to emphasize the use of the potential of ΑΙ within these applications, which will lead to the maximization of the associated benefits for women with gestational diabetes and their neonates. Furthermore, it is important to emphasize the methodological quality of the studies, since their scores on the Jadad Scale ranged from moderate to low. While double-blinding may be challenging or even impossible, emphasis should be given to other critical parameters, such as the consistent reporting of withdrawals and dropouts. Finally, the apps should also include psychological support, since the increased awareness of the risks of GDM might lead to distress.
